# Design and Performance of an Adsorption Bed with Activated Carbons for Biogas Purification

**DOI:** 10.3390/molecules27227882

**Published:** 2022-11-15

**Authors:** Giulia Molino, Marta Gandiglio, Sonia Fiorilli, Andrea Lanzini, Davide Drago, Davide Papurello

**Affiliations:** 1 Department of Energy (DENERG), Politecnico di Torino, Corso Duca degli Abruzzi, 24, 10129 Turin, Italy; 2 Department of Applied Science and Technology (DISAT), Politecnico di Torino, Corso Duca degli Abruzzi, 24, 10129 Turin, Italy; 3 Energy Center, Politecnico di Torino, Via Borsellino 38/18, 10129 Turin, Italy

**Keywords:** biogas, adsorption, purification, SOFC

## Abstract

Organic waste can be efficiently converted into energy using highly efficient energy systems, such as SOFCs coupled to the anaerobic digestion process. SOFC systems fed by biogenous fuels, such as biogas or syngas, suffer long-term stability due to trace compound impacts. It follows that, a mandatory gas cleaning section is needed to remove these pollutants at lower concentrations. This work investigates the adsorption mechanism for micro-contaminant removal through experimental results achieved using solid sorbents. Samples of different sorbent materials were analyzed in the laboratory to determine their performances in terms of sulfur (mainly hydrogen sulfide) and siloxanes (mainly D4-Octamethylcyclotetrasiloxane) adsorption capacities. The analysis shows that the chemical composition of the samples influences the adsorption of H_2_S (i.e., presence of calcium, iron, copper), while the effect of their textural properties mainly influences the adsorption of siloxane compounds, such as D4. A quantitative analysis was performed considering the influence of gas velocity on adsorption capacity. By increasing the biogas velocity (+45% and +89%), there was an indirect correlation with the H_2_S adsorption capacity (−27% and −44%). This identified an aspect related to the residence time required to be able to remove and retain the trace compound. The results obtained and summarized were used to develop a strategy for the removal of trace compounds in large-scale plants, e.g., for water purification.

## 1. Introduction

In the context of the clean energy transition, biogas is a renewable source that can be obtained by the anaerobic digestion of organic waste, such as sewage, animal or food waste, agricultural residues and the organic fraction of municipal solid waste [1]. The waste-to-biogas conversion occurs in an anaerobic digester in which bacteria convert organic matter into anaerobic digester gas—ADG [2,3]. ADG mainly contains methane and carbon dioxide, and it is commonly used to produce electricity in internal combustion engines (ICEs), for which the literature reports an average efficiency of 37% [4]. A more efficient technology for electricity generation from ADG is the fuel cell (FC) which is an electrochemical device that directly converts chemical energy into electric energy without combustion. In particular, high-temperature fuel cells, molten carbonate and solid oxide can be feed directly with ADG producing electricity with electrical efficiencies around 45–47% for MCFC and higher than 50% for SOFC [5]. Unlike combustion in ICEs, FCs do not release atmospheric pollutants (e.g., particulate matter, NOx, SOx, or VOC) into the atmosphere. On the other hand, ADG initially contains a wide range of micro-contaminants that have a detrimental effect on the FC anode, causing a performance drop causing fatal degradation [6,7,8,9,10,11]. Therefore, a gas processing unit (GPU) or clean-up unit is required to eliminate these substances [12,13,14,15]. The most common contaminants are hydrogen sulfide (H_2_S) [16,17] and sulfur compounds [18,19], and siloxanes [20] which are molecules that contain silicon linked with oxygen and halogens (HCl and HF) [21,22]. Moreover, many other contaminants are contained in the biogas, as reported in papers [23,24,25,26] and databases [27]. Worldwide there are a few demonstration plants that generate electricity through ADG fuel cells [28,29,30], including the one deployed in DEMOSOFC, an EU project started in 2015. This plant is installed in the SMAT Collegno waste-water treatment plant—WWTP—and it is the first European industrial-sized biogas-fed solid oxide fuel cell (SOFC) plant [31].

Contaminant removal from biogas can be obtained in different ways; the first distinction among the various methods is during or after the digestion: during digestion, it can be obtained by injecting substances directly inside the digester (in situ), especially for sulfur reduction [32], whereas after digestion it can be achieved through absorption [14,33], membrane processes [14,34] or dry processes. Among the dry processes, it occurs via the adsorption process which is the only technique that can reduce the concentration of contaminants to the “ultra-low sulfur level needed for fuel cell feeding” [8,34,35,36]. The adsorption process can be obtained with selected materials: zeolites, metal oxides, natural or impregnated carbons [14] and also silica gel [33]. In ADG fuel cell demonstration plants, often a multi-step GPU is present in combination with different cleaning methods, frequently with a pre-treatment stage; moreover, the GPU can change depending on the maximum level of different hazardous compounds, since biogas contaminant concentrations can vary from one WWTP to another. Adsorption through impregnated carbons is a cleaning method that can be found in many ADG fuel cell demonstration plants [29,30,37,38]. Bak et al., (2019) demonstrated how the iron content affects sulfur removal. The micropore distribution influences the retention of heavier compounds, such as CS_2_ [38]. De Arespacochaga et al., (2015) proved in a real plant how effective deep cleaning is in ensuring the continuous operation of a SOFC stack for more than 700 h. This cleaning system consisted of three beds in series, with iron-based materials, a temperature control to condense the trace compounds, and an activated carbon bed [30].

This work presents a novel experimental approach to assess the adsorption capacity of activated carbons (ACs) with biogas contaminants. The authors investigated the adsorption mechanism for the removal of contaminants from ADG removal using a bed of ACs as the main process for the biogas clean-up unit of an FC plant. The goal of the work was to identify the relationship between adsorption capacity and the physicochemical properties of AC by experimentation. The novelty of the presented work relies on the integration of different material analyses and rigorous experimental work. A quantitative analysis was performed considering the influence on adsorption capacity with gas velocity.

Furthermore, commercial sorbents were evaluated and the results were used for a real application: the DEMOSOFC plant, the first industrial-sized biogas-fed SOFC system currently running.

Samples of different ACs were analyzed by physicochemical characterization techniques and tested in the laboratory to determine their performance in terms of H_2_S and D4-siloxane adsorption capacities. H_2_S and D4 have been chosen since they are the most abundant in this type of biofuel [15,39].

The analysis of experimental data allowed to extrapolate the relationships between the physicochemical properties (e.g., specific surface area, micropore volume, total pore volume, metal oxide atomic concentration) of the samples and their adsorption capacity. The relationships found enabled determination of the adsorption capacity behavior of a new sample without experimental evaluation of the breakthrough curve.

## 2. Materials and Methods

This section is related to the analysis of the correlation between the selected sample’s textural properties measured at the DISAT—Applied Science and Technology Department (Polytechnic of Turin, IT)—laboratory and the sample’s adsorption capacity obtained through experimentation performed at DENERG—Energy Department (Polytechnic of Turin, IT)—laboratory and at the Edmund Mach Institute (Trento, IT).

### 2.1. Materials

Five selected commercial materials for micro-contaminants adsorption were analyzed to determine the performances of the sample in terms of H_2_S and D4 adsorption capacities:

Three steam activated carbons, CKC, CKI, C64 (AirDep [40]);
One activated carbon with dispersed metal oxides, R8G (SulfaTrap [41]);
One metal oxide, R7E (SulfaTrap).

CKC is a carbon of mineral origin extruded to obtain small cylinders with a particle diameter of 4 mm, steam activated, and impregnated with potassium bicarbonate at 5%. CKI is a carbon of mineral origin extruded to obtain small cylinders with a particle diameter of 4 mm, steam activated, and impregnated with potassium iodide at 2%. C64 is a carbon of mineral origin extruded to obtain small cylinders with a particle diameter of 4 mm, and steam activated with an alkaline pH. CKC and CKI are suggested by the producer for sulfur removal, while C64 is recommended for siloxane removal.

R8G is an activated carbon with a highly dispersed mixed metal oxide active phase with modifiers prepared over a porous support [42].

R7E is a metal oxide that contains copper oxide. The information on the composition was not available from the producer but was obtained through energy dispersive X-ray spectroscopy (EDS) at the DISAT laboratory. This sample also contained manganese, aluminum and silicon (detailed results are presented in the Appendix A). R8G and R7E are both recommended for sulfur removal from biogas.

### 2.2. Evaluation of Textural Properties

A gas sorption analyzer Quantachrome Autosorb 1 (Boynton Beach, FL, USA) was adopted for the determination of adsorption isotherms for N_2_ at 77 K. Samples were outgassed at 423 K overnight before the adsorption measurements. The experimental equipment allows measurement of the relative pressure until 10^−6^ p/p_0_. Micropore volumes were determined using the *t-Plot* method in the relative pressure range of 0.15–0.3. For carbon-based materials, the pore size has been evaluated through the DFT method (density functional theory), using the NLDFT (nonlocal density functional theory) equilibrium model for slit/cylindrical pores. The BET method is “the most widely used procedure for evaluating the surface area of porous materials” [43].

The *t-Plot* method determines the presence of micropores in a solid by comparing the material adsorption isotherm with a reference one, specific for the material under investigation. This method allowed to determine the micropore volume—*V micropore* (cm^3^/g)—of the samples.

The DFT method provides a “reliable approach to pore size analysis over the complete nanopore range” (up to 100 nm); there are different pore shape models developed for various material classes, such as carbons [43]. The total pore volume—*Pore volume* (cm^3^/g)—of the samples was obtained through this method.

A scanning electron microscopy (SEM) (FEI Inspect, Philips 525 M) coupled with EDS analysis (SW9100 EDAX) was adopted to characterize the sorbent samples. The localized chemical analysis was achieved by EDS (energy dispersive X-ray spectrometry). Qualitative analysis was achieved through the identification of the lines reported in the spectrum, while quantitative analysis (determination of the percentage elemental composition) was performed through the measurement of line intensities for each element present in the sample. The results of this analysis are presented in the Appendix A.

**Figure 1:**
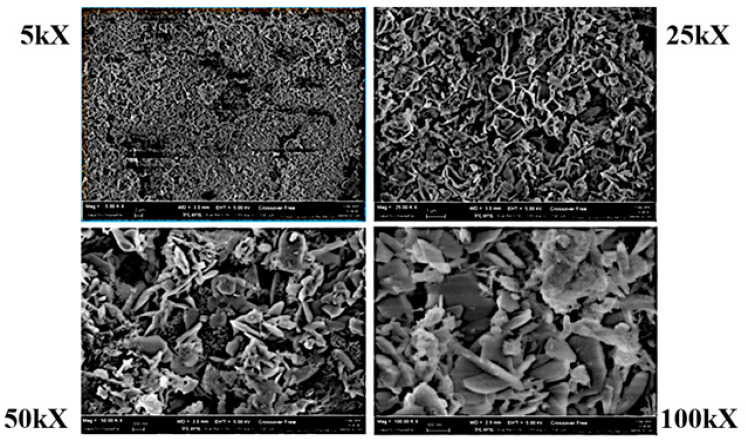
R8G SEM images.

Figure 1 shows a series of SEM images of R8G with increasing magnification: 5000, 25,000, 50,000, 100,000 times. The SEM images illustrate the porous structure of the AC, highlighting the presence of discontinuity in the sample’s surface. The presence of high porosity is necessary to limit the energy losses from the blower system to filter the biogas. As reported in some of our previous studies, SEM images coupled with an EDS analysis can be useful to highlight the sulfur adsorption and retention within the pores and metal-based catalytic sites [15,44].

### 2.3. Evaluation of the Adsorption Capacity

Laboratory tests were performed to evaluate the adsorption capacity of the chosen materials. The tests were performed using three different reactors filled with carbon:

Microreactor 4 mm: the first set of experiments was made using micro-reactors with an internal diameter of 4 mm, filled with ground carbon, sieved to select particles with size of 50 µm–70 µm; the carbon retainment was obtained with medical gauze. Closing of the reactor was obtained with a nut and washer made of steel with sulfur-inert coating.

Large reactor: the last set of experiments was made using a reactor constructed in the laboratory, with an internal diameter of 25 mm, filled with carbon as-received from the supplier; carbon blocking was obtained through using cotton wool.

The reactors were fed with simulated biogas from cylinders: the biogas composition was chosen based on average values from measurements performed at the DEMOSOFC site: 62.5 %vol. methane and 37.5 %vol. carbon dioxide. Hydrogen sulfide (H_2_S) was fed into the system using methane cylinders with the fixed concentration of the contaminant (usually between 100 and 1000 ppmv).

The sensor used for the detection of hydrogen sulfide was an electrochemical device that could measure from 0 to 200 ppmv (Transmitter MECCOS eTR H_2_S 0–200 ppmv, Siegrist GmbH, Karlsruhe, DE); the error of measurement increases linearly from 0 to 200 ppmv at full scale. To find the percent error, first, we averaged all our measurements. Then, we found the difference between our average and true values. The sensor has flow limits between 500 mL/min and 1000 mL/min, so all the experiments were performed considering this flow range. In all published manuscripts, authors refer to the volumetric flow rate defined under normal conditions. A calibrated sensor was used to verify the value of the measured H_2_S level, calculating also the signal delay. Compressed air was used to purge the sensor at the end of the tests.

**Figure 2:**
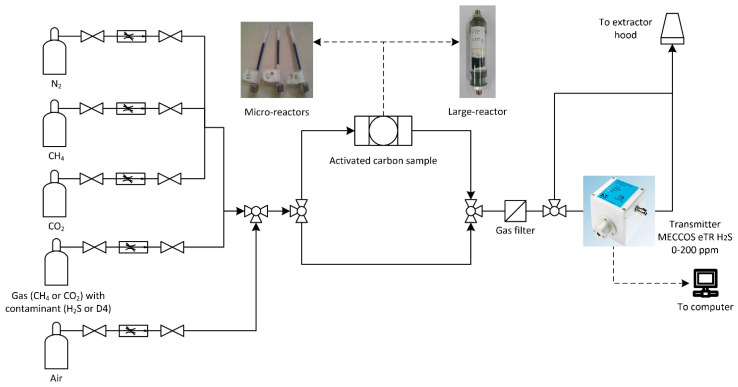
The layout of the experimental apparatus.

The aim of these experiments was to calculate the quantity of contaminant that was adsorbed by the carbon filters, using the experimental apparatus presented in Figure 2. The experiments allow the determination of the adsorption curve, which is the evolution of the contaminant concentration over time at the outlet of the fixed bed. This concentration will rise, following a certain trend, from zero (when the filter is completely removing the contaminant) to the inlet contaminant value (saturation, when the sorbent is no longer filtering any contaminants and the outlet concentration is equal to the inlet one). The area below the adsorption curve is the amount of contaminant adsorbed by the carbon material. From this knowledge, the adsorption capacity *Ads Cap* (mg/g), defined as an evaluator of the sorbent’s performance, can be calculated.

The adsorption capacity can be typically evaluated at breakthrough or saturation.

Breakthrough adsorption capacity: This value refers to the moment when the concentration of the contaminant after the filter reaches the C/C_0_ fixed threshold value (usually 1% in the literature), where C (mol/m^3^) is the actual H_2_S concentration in the gas phase and C_0_ (mol/m^3^) is the H_2_S inlet concentration. The C/C_0_ ratio is fundamental and should be set accurately when the cleaning system aims to protect a device downstream of the cleaning section (for example a fuel cell system that could be seriously damaged by contaminants at very low levels, <1 ppmv). The adsorption performance is influenced by the slope of the saturation curve profile. If the slope is shallow, the saturation is reached earlier even if the ideal linear front is far from the bed outlet. The length of the mass-transfer zone (MTZ), where adsorption takes place [45], is responsible of the breakthrough time.

Saturation adsorption capacity: This value is related to the maximum value of adsorbed contaminant into the filter; it is evaluated at the moment when the amount of contaminant at the exit of the bed is the same as the inlet value (C = C_0_). This value could be useful in case a series of beds are available in the system. In this case, the first cleaning vessel could be operated even after breakthrough and until saturation (to fully exploit the material) because other beds in series are available.

If the slope of the curve is steep, the difference between these two adsorption capacities is low. In the experiments, we fixed the breakthrough concentration limit for the fuel cell feeding and evaluated the adsorption capacity at this breakthrough condition. The adsorption capacity was evaluated as:

(1) $$Ads\;Cap_{S}=\frac{{\dot{q}}_{biogas}\cdot \frac{1}{10^{3}}\cdot \frac{1}{60}\cdot \frac{1}{22.414}\cdot C_{S}\cdot MW_{S}\cdot t_{BT}}{m_{AC}}$$

where ${\dot{q}}_{biogas}$ is the biogas volumetric flow rate used for the experiment $\left(\frac{ml}{min}\right),$ *C_S_* is the sulfur volumetric concentration $\left(\frac{vol_{S}}{vol_{biogas}}\right)$, *MW_S_* is the molecular weight of sulfur (g/mol), *t_BT_* (s) the breakthrough time (retrieved from the experimental adsorption curve) and *m_AC_* is the mass of sorbents contained in the reactor.

The time considered in the formula—*t_BT_* (s)—is the time at which there is a breakthrough, defined as the moment in which the concentration downstream of the filter starts to be different from zero. The breakthrough time was measured considering the value of the outlet concentration measured by the sensor. This was determined as the first point of the outlet concentration measured by the sensor that was higher than zero.

### 2.4. Design of Experiments

Table 1 reports the experimental plan. The R7E and C64 powders were not analyzed with the microreactor because it was not possible to perform the analysis with the ground samples due to the excessive pressure drop induced by the small size (50–70 µm) of ground carbon, which was too compact for the fixed biogas flow rate.

## 3. Results and Discussion

### 3.1. Textural Properties Effect

The first step was to analyze the correlation between the surface area and pore volume of the materials with trace compound adsorption capacities. A good correlation was obtained for the siloxane D4 adsorption capacity (Table 2 summarizes D4 data), whereas these parameters did not influence the adsorption of H_2_S at ambient temperatures. Therefore, the second step was to find a link between the AC’s surface chemical composition and the H_2_S adsorption capacity, based on the traces of some metals contained inside the ACs. The textural analysis of the samples allowed the interesting relationships between the physicochemical properties of the samples and their adsorption capacity to be obtained: the effect of samples structural properties influences especially D4 adsorption, whilst the samples chemical composition influences the H_2_S adsorption. D4 adsorption is more influenced by the physical structure rather than the chemical composition because it is a neutral molecule and its size is bigger than H_2_S [46]; on the other hand, H_2_S is acidic and its adsorption is characterized by the interaction with the sample’s chemical surface composition [47].

#### 3.1.1. D4 Adsorption Capacity Correlations

Table 2 shows the data referring to the textural properties of the ACs. The adsorption capacity of the siloxane D4 was obtained through the extension of experimental results obtained at the Edmund Mach Institute located in San Michele (TN) with the same method explained in the paragraph “Adsorption capacity calculation” [48].

**Figure 3:**
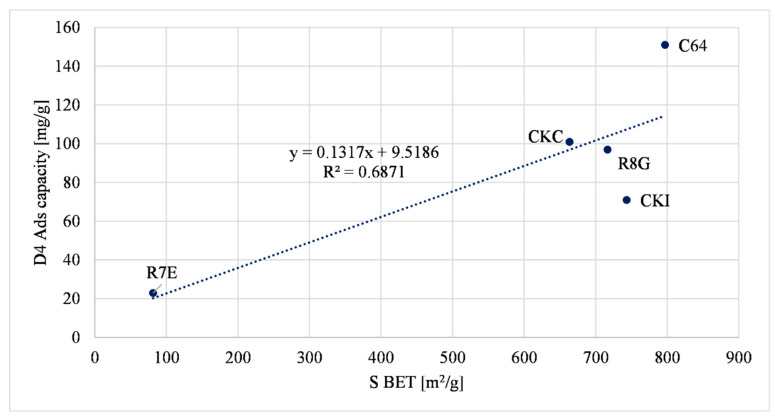
S BET correlation with D4 adsorption capacity.

Figure 3 illustrates the linear correlation between the S BET specific surface area of carbons versus the siloxane (D4) adsorption capacity. The R7E material shows a lower surface area value compared to the other samples. This is due to the highest content of metal oxides inside the sample.

**Figure 4:**
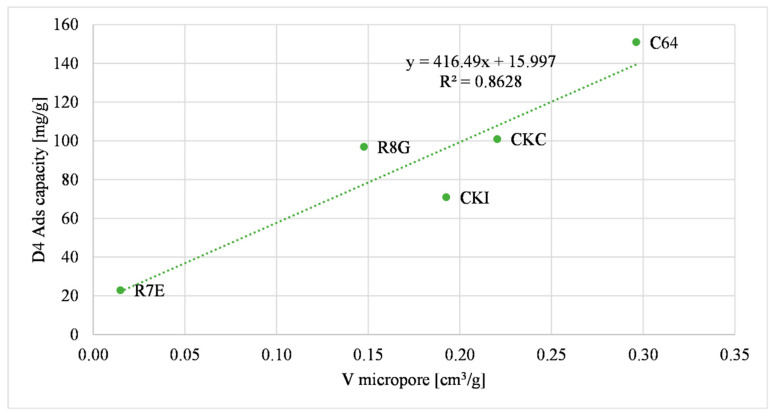
Micropore volume correlation with D4 adsorption capacity.

Figure 4 shows the linkage between the adsorption capacity of siloxane D4 and the micropore volume of the activated carbons (<2 nm).

**Figure 5:**
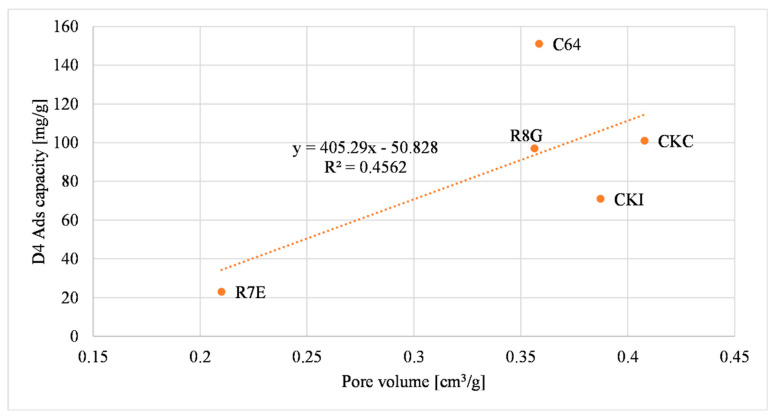
Total pore volume correlation with D4 adsorption capacity.

Figure 5 illustrates the siloxane D4 adsorption capacity related to the total pore volume of the materials.

In Figure 3, Figure 4 and Figure 5 a direct relationship between some of the physical properties and the D4 adsorption capacity is shown. The main adsorption phenomenon related to D4 is the “physisorption”, or the “physical adsorption”, a phenomenon characterized by the van der Waal interaction between the adsorbed gas and the surface of the sample. Similar considerations were assessed by other research studies that considered D4 as a gas pollutant [46,49].

The parameter R^2^ was chosen to compare the effect of the physical properties of the samples. Considering these data, the micropore volume was the parameter that had the greatest influence on the D4 adsorption capacity because the R^2^ was the highest value among the other three (it is equal to 0.8628). The S BET specific surface area of the materials had a lesser influence on the D4 adsorption capacity compared to the micropore volume, considering that the R^2^ value was lower (i.e., equal to 0.6871). The total pore volume of the materials was the parameter that had the lowest R^2^ value (i.e., equal to 0.4562), so it had the least influence on the D4 adsorption capacity. These results were compared with the reported literature data [50]. Cabrera-Codony et al., (2014) showed the influence of selected physical characteristics on D4 adsorption. The micropore volume, the S BET specific surface area and the total pore volume were considered. The total pore volume had the biggest influence on the D4 adsorption capacity. Increasing the number of samples tested may provide a better correlation between the total pore volume and the D4 adsorption capacity.

Considering the data regressions, it is possible to compare the selected physical properties of a new sample with experimental data and presume the value of the D4 adsorption capacity of the new sample.

Results achieved here demonstrate that the adsorption capacity for volatile siloxane content in biogas is mainly related to physical features rather than chemical composition, as reported also by Schweigkofler and Niessner [33] and Matsui and Imamura [51].

#### 3.1.2. H_2_S Adsorption Capacity Correlations

The relationship between H_2_S adsorption capacity and ACs surface chemical composition was investigated through analyzing the type of metal oxides found by EDS analysis. In this case, the adsorption is called “chemisorption”, where the intermolecular forces involved lead to the formation of chemical bonds [43]. The reactions that occur between H_2_S and metal oxides on the surface of the sample are of acid–base type.

Two sets of data were obtained from the experimental tests described above. The first set is related to AC powder (carbons that were previously ground) inside the microreactor, the other set refers to AC as received inside the large reactor. The adsorption capacities obtained from the activated carbons as received were considered more reliable because the grain size is not modified with the surface features. Therefore, the EDS analysis was performed on the carbon sample as received from the supplier.

Table 3 shows the results obtained from the EDS analysis, expressed as percentages of atomic concentration; the remaining percentage of each sample is carbon. It can be seen that R7E did not contain carbon but metal oxides, such as CuO, Al_2_O_3_ and MnO_2_.

These results are an average of the analyses performed on different regions of the same sample. Data from different regions obtained with the EDS analysis are reported in detail in Table A1 in the Appendix A paragraph.

##### Microreactor Data: AC Powders

This section is related to the H_2_S adsorption capacities obtained through experiments performed with the microreactor filled with ground activated carbons.

The experiments were performed at ambient temperatures with a flow rate equal to 750 mL/min, a H_2_S concentration of 20 ppmv and a biogas velocity of 0.995 m/s. Table 4, shows the H_2_S adsorption capacity values obtained in the laboratory and the atomic concentration percentages of metals that influence the chemisorption. The R7E and C64 are not reported because it was not possible to perform the analysis with the ground sample. This is due to the compactness of R7E and C64 powders. It follows that biogas could not flow inside such samples due to the limited porosity.

**Figure 6:**
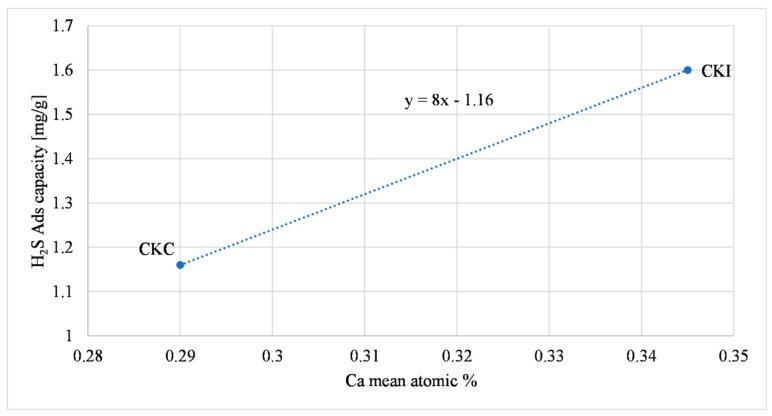
Calcium oxide effect on H_2_S adsorption.

The following comparison refers to carbons which contain calcium oxide, CKC and CKI; the increment of calcium oxide was directly proportional to H_2_S adsorption capacity, as presented in Figure 6; Equation (2) describes a possible reaction with H_2_S, described also in Agnihotri et al., (1999) [52].

(2) $$CaO+H_{2}S\to CaS+H_{2}$$

**Figure 7:**
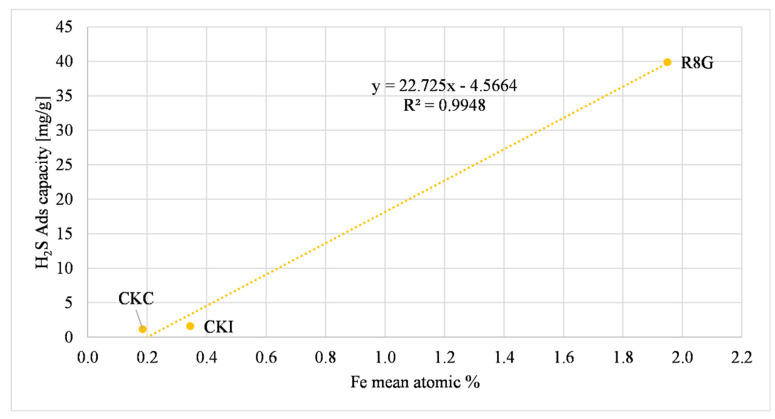
Iron effect on H_2_S adsorption.

Another metal that can influence the H_2_S adsorption capacity is iron. A possible explanation of this relationship is a redox reaction, as reported in de Arespacochaga et al., (2014) [34]. The relation described can be summarized by a two-step equation, Equations (3) and (4). Figure 7 shows the direct proportional relationship between iron and the H_2_S adsorption capacity.

(3) $$2Fe{\left(OH\right)}_{3}+H_{2}S\to 2Fe{\left(OH\right)}_{2}+2H_{2}O+S_{\left(S\right)}$$

(4) $$2Fe{\left(OH\right)}_{2}+2H_{2}S\to 2FeS+4H_{2}O$$

This mechanism of removal was also underlined in other research papers [19,41].

The R8G has an iron atomic concentration that is higher than the other ACs (one order of magnitude), which may influence the H_2_S adsorption capacity that was considerably higher than in the other samples.

Overall it can be seen that some metal oxides found out with EDS analysis (calcium and iron oxides) affect the H_2_S adsorption capacity of ground samples, this is supported by previous studies [53,54,55].

##### Large Reactor Data: AC As-Received

This section is related to data obtained in the laboratory with the large reactor filled with the ACs as received (with pellet shape). The experiments were performed at ambient temperatures with a flow rate equal to 950 mL/min, a H_2_S concentration of 20 ppmv and a biogas velocity of 0.032 m/s. Table 5, shows the H_2_S adsorption capacity values obtained in the laboratory and the atomic concentration percentages of metals that influence the chemisorption.

**Figure 8:**
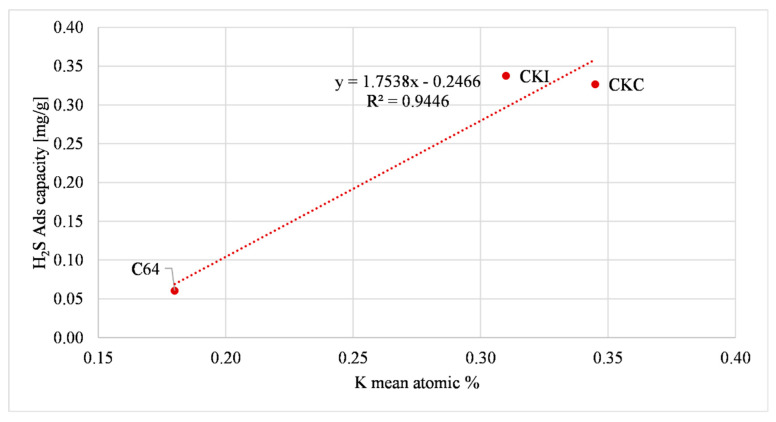
Potassium oxide effect on H_2_S adsorption (AC as received).

The first relationship refers to carbons that contain potassium, CKC, CKI and C64. Figure 8 shows the effect of the presence of potassium on H_2_S adsorption. The figure shows that CKC and CKI, which contain almost double quantity of potassium compared to C64, had an H_2_S adsorption capacity that was more than five times bigger.

**Figure 9:**
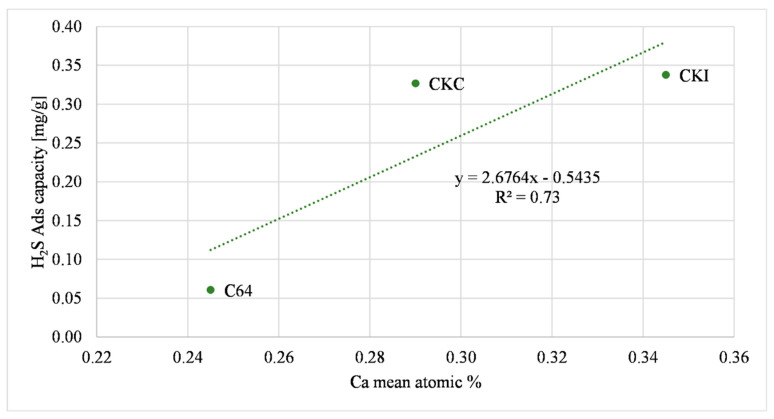
Calcium oxide effect on H_2_S adsorption (AC as received).

The influence of calcium on the H_2_S adsorption capacity shown in Figure 6 with ground samples was also discerned with AC as received. Figure 9 shows that the increase in calcium oxide was directly proportional to the H_2_S adsorption capacity, as supported by previous studies [56,57]. A possible relationship that describes this behavior is shown in Equation (2).

**Figure 10:**
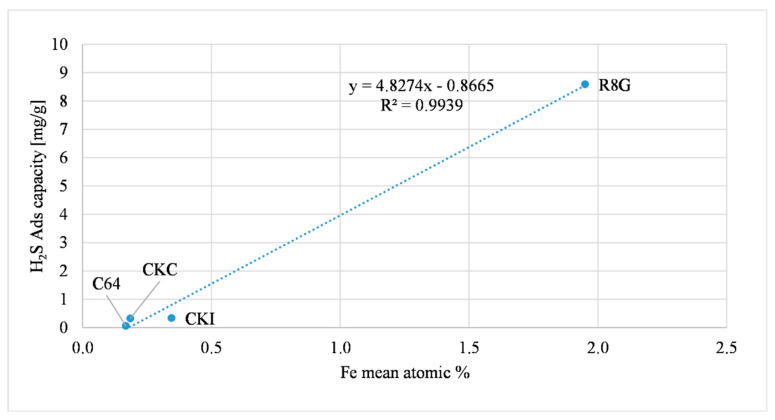
Iron effect on H_2_S adsorption (AC as received).

The influence of iron on the H_2_S adsorption capacity was discerned with AC as received. Figure 10 represents this relation. This trend was comparable to Figure 7, referred to as the microreactor filled with ground AC. As reported before, the R8G sample had an iron atomic concentration that was higher than the other ACs (one order of magnitude), this influenced the H_2_S adsorption capacity increasing the magnitude of adsorption. A possible redox equation is presented in Equations (3) and (4). The adsorption capacity of the R8G sample was sensibly lower compared to the ground R8G sample, due to a possible inhomogeneity with small reactor tests caused by the very small amount of ground sample (0.15 g).

**Figure 11:**
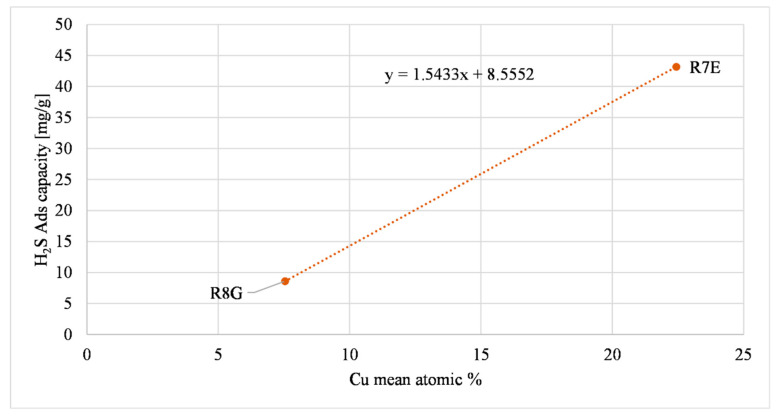
Copper oxide effect on H_2_S adsorption (AC as-received).

The last relationship refers to carbons that contained copper, R8G and R7E. Figure 11 illustrates the influence of copper on H_2_S adsorption. The H_2_S adsorption capacities of R8G and R7E were significantly higher than the other tested ACs, and these two samples were the only ones that contained copper, so the presence of copper enhanced the H_2_S chemisorption at ambient temperatures. A possible reaction that occurs is presented in Equation (5).

(5) $$CuO+H_{2}S\to CuS+H_{2}O$$

This mechanism is supported by Calbry-Muzyka et al., (2019) [58]. The R7E copper atomic concentration was three times bigger than R8G and affected the H_2_S adsorption capacity which was five times bigger than R8G. The R7E adsorption capacity was higher also because the L/D of the R7E test was higher than the L/D of the R8G test, due to the greater amount of sample inside the reactor (as explained in Table 1). The L/D value affects the H_2_S adsorption as demonstrated in a previous paper [14].

Overall, it was demonstrated that some metal oxides identified by EDS analysis (i.e., potassium, iron and copper oxides) affect the H_2_S adsorption capacity of samples as-received (with pellet shape).

As a qualitative result, it can be seen that the presence of copper (chemisorption mechanisms) has a bigger influence on the H_2_S adsorption capacity at ambient temperatures, with R8G and R7E, which contain copper, having the highest H_2_S adsorption capacity.

The chemisorption reactions for the sulfur compounds were stronger than the physisorption reactions, as highlighted by Lee et al., (2017) [59]. Chemisorption reactions involving CaO, CuO, Fe(OH)_2_ and Fe(OH)_3_ are described in Equations (2) to (5).

## 4. Conclusions

This paper focused on the physical phenomena that describe the performance (in terms of adsorption capacity) of sorbent materials for the removal of biogas micro-contaminants. Among all the contaminants, H_2_S and siloxanes were selected for the experimental analysis, because of their higher concentrations in biogas and their significant detrimental effect on fuel cells.

The AC’s surface chemical composition influenced the H_2_S adsorption capacity. CKC, CKI and C64 were mostly influenced by the presence of calcium oxide and potassium oxide inside their chemical composition. R8G and R7E were strongly influenced by the copper oxide content in their structure. The H_2_S adsorption capacity of all analyzed samples (except R7E) was influenced by the presence of iron. The ACs textural properties influenced the D4 adsorption. Based on our analysis, the micropore volume was the parameter that had the greatest influence on the D4 adsorption capacity. It can be summarized that the chemical composition of a sample influences the H_2_S adsorption, whilst the effect of their textural properties influences the adsorption of D4 in particular. A quantitative analysis was performed considering the influence of gas velocity on the adsorption capacity. Increasing the biogas velocity (+45% and +89%) there was an indirect correlation with the adsorption capacity of H_2_S (−27% and −44%).

The results obtained and summarized were used to develop a strategy for the removal of trace compounds in large-scale plants, e.g., for water purification. The chosen strategy was to insert different beds in series for the removal of specific compounds, e.g., sulfur compounds and then silicon compounds. Further details on this can be found on the web (https://demosofc.wordpress.com/ (accessed on 10 November 2022).

## Figures and Tables

**Table 1:** Design of the experiments. * The R7E pellets were very irregular, so packing was not guaranteed with 7.5 g only because there were paths among the pellets. This caused preferential routes that led to a decreased filter performance. To solve this problem, the length of the filter was increased raising also the sample amount. The final R7E sample weight was 11.7 g. ** Normal conditions.

	Test Set #1	Test Set #2	Test Set #3	Test Set #4
**Reactor type**	Microreactor 4 mm	Large reactor	Microreactor 4 mm	Microreactor 4 and 10 mm
**Experiment**	Sulfur	Sulfur	Siloxane	Sulfur, speed experiment
**Samples**	CKC, CKI, R8G	C64, CKC, CKI, R8G, R7E	C64, CKC, CKI, R8G, R7E	R8G
Flow rate **	750 mL/min	950 mL/min	200 mL/min	500–750 mL/min
**Gas composition**	62.5% CH_4_–37.5% CO_2_	62.5% CH_4_–37.5% CO_2_	62.5% CH_4_–37.5% CO_2_	62.5% CH_4_–37.5% CO_2_
**Contaminant**	H_2_S	H_2_S	D4	H_2_S
**Contaminant concentration**	20 ppmv	20 ppmv	20 ppmv	20 ppmv
**Gas linear velocity**	0.995 m/s	0.032 m/s	0.27 m/s	Range 0.1–1 m/s
**Pression**	Ambient pressure	Ambient pressure	Ambient pressure	Ambient pressure
**Temperature**	Ambient temperature	Ambient temperature	Ambient temperature	Ambient temperature
**Sample weight inside filter**	0.15 g ± 0.01 g	7.5 g ± 0.5 g 11 g ± 0.5 g *	0.15 ± 0.05 g	0.07–0.92 g
**GHSV**	154.8–190.8 kh^−1^	2520–6624 h^−1^	20.2–35.36 kh^−1^	20.10–325.54 kh^−1^

**Table 2:** Textural properties of ACs.

Sample	Micropore Volume (cm^3^/g)	Total Pore Volume (cm^3^/g)	Surface Area (m^2^/g)	D4 Ads. Capacity (mg/g)
	t-Plot	DFT	BET	
R8G	0.15	0.36	716.5	97 ± 3.1
R7E	0.015	0.21	81.5	23 ± 1.1
C64	0.3	0.36	796.8	151 ± 5.3
CKC	0.22	0.41	663.4	101 ± 3.8
CKI	0.19	0.39	743.2	71 ± 2.4

**Table 3:** Atomic concentration percentage composition of the samples (EDS).

Sample	O	Si	Al	K	Ca	Fe	S	Mg	Na	Pt	I	Ti	Cu	Mn
**C64**	10.77	0.46	0.38	0.18	0.25	0.17	0.12	0.09	0.04	0.10				
**CKC**	11.32	0.48	0.38	0.35	0.29	0.19	0.15	0.09						
**CKI**	7.70	0.44	0.43	0.31	0.35	0.35	0.71	0.09			0.27	0.05		
**R8G**	42.58	0.41	0.26			1.95	4.14						7.54	
**R7E**	60.89	4.30	5.865										22.43	6.515

**Table 4:** Atomic concentration percentages and microreactor data.

Sample	Ca Atomic Concentration	Fe Atomic Concentration	H_2_S Ads Capacity Microreactor (mg/g)
**CKC**	0.29	0.19	1.16 +/− 0.05
**CKI**	0.35	0.35	1.60 +/− 0.06
**R8G**	-	1.95	39.9 +/− 1.2

**Table 5:** Atomic concentration percentages and large reactor data.

Sample	K	Ca	Fe	Cu	H_2_S Ads Capacity Large Reactor (mg/g) (AC as Received)
C64	0.18	0.25	0.17	-	0.06 +/− 0.005
CKC	0.35	0.29	0.19	-	0.33 +/− 0.01
CKI	0.31	0.345	0.35	-	0.34 +/− 0.015
R8G	-	-	1.95	7.54	8.59 +/− 0.4
R7E	-	-	-	22.43	43.17 +/− 1.4

## Data Availability

Not applicable.

## Abbreviations

AC	Activated Carbons
ADG	Anaerobic Digester Gas
Ads Cap	Adsorption capacity
BET	Brunauer Emmett Teller method
CKC	AirDep CKC activated carbon of mineral origin
D4	Octamethylcyclotetrasiloxane
DFT	Density Functional Theory
EDS	Energy-dispersive X-ray spectroscopy
FC	Fuel Cell
GHG	Greenhouse Gas
GPU	Gas Processing Unit
ICE	Internal Combustion Engine
MCFC	Molten Carbonate Fuel Cell
MTZ	Mass Transfer Zone
NLDFT	Nonlocal Density Functional Theory
SEM	Scanning Electron Microscopy
SOFC	Solid Oxide Fuel Cell
VOC	Volatile Organic Compounds
WWTP	Waste-Water Treatment Plant
**Nomenclature**	
C is the H_2_S concentration in the gas phase, $\frac{mol}{m^{3}}$	
$C_{0}\;$ is the H_2_S inlet concentration, $\frac{mol}{m^{3}}$	
$Ads\;Cap_{S}$ is the sulfur adsorption capacity, $\frac{g_{S}}{g_{AC}}$	
${\dot{q}}_{biogas}$ is the biogas volumetric flow, $\frac{Nml}{min}$	
$C_{S}$ is the sulfur volumetric concentration, $\frac{vol_{S}}{vol_{biogas}}$	
$MW_{S}$ is the sulfur molecular weight, $\frac{g}{mol}$	
$t_{BT}$ is the breakthrough time, s	
$m_{AC}$ is the weight of AC contained inside the sample, g	

## Appendix A

**Table A1:** EDS analysis results of the samples. The atomic concentration is a percentage, the remaining percentage of each sample is composed by carbon.

		Atomic Concentration (EDS)													
**Sample**	Region	**O**	**Si**	**Al**	**K**	**Ca**	**Fe**	**S**	**Mg**	**Na**	**Pt**	**I**	**Ti**	**Cu**	**Mn**
**C64**	1	11.37	0.47	0.4	0.33	0.29	0.18	0.15	0.08	0	0				
	2	11.27	0.48	0.36	0.36	0.29	0.19	0.15	0.09	0	0				
	3	11.68	0.37	0.32	0	0.18	0.16	0.07	0.06	0.05	0				
	4	8.77	0.50	0.44	0.03	0.22	0.14	0.11	0.12	0.10	0.41				
	**mean value**	**10.77**	**0.46**	**0.38**	**0.18**	**0.25**	**0.17**	**0.12**	**0.09**	**0.04**	**0.10**				
**CKC**	region														
	1	11.37	0.47	0.4	0.33	0.29	0.18	0.15	0.08						
	2	11.27	0.48	0.36	0.36	0.29	0.19	0.15	0.09						
	**mean value**	**11.32**	**0.475**	**0.38**	**0.345**	**0.29**	**0.185**	**0.15**	**0.085**						
**CKI**	region														
	1	6.44	0.54	0.33	0.39	0.63	0.48	0.63	0.05			0.4	0.06		
	2	8.96	0.34	0.52	0.23	0.06	0.21	0.79	0.13			0.14	0.03		
	**mean value**	**7.7**	**0.44**	**0.425**	**0.31**	**0.345**	**0.345**	**0.71**	**0.09**			**0.27**	**0.045**		
**R8G**	region														
	1	39.1	0.49	0.26			2.79	4.5						5.62	
	2	46.05	0.32	0.26			1.11	3.77						9.46	
	**mean value**	**42.575**	**0.405**	**0.26**			**1.95**	**4.135**						**7.54**	
**R7E**	region														
	1	61.32	3.71	5.69										22.87	6.4
	2	60.46	4.88	6.04										21.99	6.63
	**mean value**	**60.89**	**4.295**	**5.865**										**22.43**	**6.515**
